# Medulloblastoma with Excessive Nodularity: Radiographic Features and Pathologic Correlate

**DOI:** 10.1155/2012/310359

**Published:** 2012-10-22

**Authors:** L. A. Yeh-Nayre, D. M. Malicki, D. N. Vinocur, J. R. Crawford

**Affiliations:** ^1^The Department of Pediatrics, University of California San Diego and Rady Children's Hospital, 3020 Children's Way, San Diego, CA 92123, USA; ^2^The Department of Pathology, University of California San Diego and Rady Children's Hospital, 3020 Children's Way, San Diego, CA 92123, USA; ^3^The Department of Radiology, University of California San Diego and Rady Children's Hospital, 3020 Children's Way, San Diego, CA 92123, USA; ^4^The Department of Neurosciences, University of California San Diego and Rady Children's Hospital, 8010 Frost St., Suite 400, San Diego, CA 92123, USA

## Abstract

Medulloblastoma with extensive nodularity is a rare subtype of the most common malignant childhood brain tumor and has been associated with more favorable prognosis. The authors report the case of a 10-month-old girl with a posterior fossa tumor of excessive nodularity with decreased diffusivity on diffusion-weighted magnetic resonance imaging sequences and robust grape-like postgadolinium contrast enhancing features. The unique neuroradiographic features were confirmed by histopathology and a diagnosis of medulloblastoma with extensive nodularity was made. This case highlights the importance of recognizing this unique medulloblastoma subtype preoperatively, as the more favorable outcome may preclude less aggressive medical management.

## 1. Introduction

Primary childhood central nervous system brain tumors occur at an incidence of 5.0 cases per 100,000/person-years in the United States according to data from the Central Brain Tumor Registry [1]. Medulloblastoma, the most common malignant tumor of childhood occurs in 0.51 per 100,000-person years and represents 13% of all childhood primary brain tumors ages 0–14 years [1]. The World Health Organization criteria still remains paramount in the classification of this grade IV malignant neoplasm. Numerous recent studies have demonstrated medulloblastoma are actually a genetically diverse group of tumors arising from the external granular layer of the cerebellum and can be classified into four distinct molecular subtypes based on gene expression microarrays [2–6]. Neuroimaging can play a role in the diagnosis of medulloblastoma, however atypical features do exist [7]. We report the case of a 10-month-old infant diagnosed with a medulloblastoma with what we term “excessive” nodularity based on neuroimaging features and confirmed by neuropathology. The specific neuroradiographic features of this medulloblastoma subtype are important to recognize as these patients have improved survival and may warrant less aggressive therapy [8, 9].

## 2. Case Report

**Figure 1:**
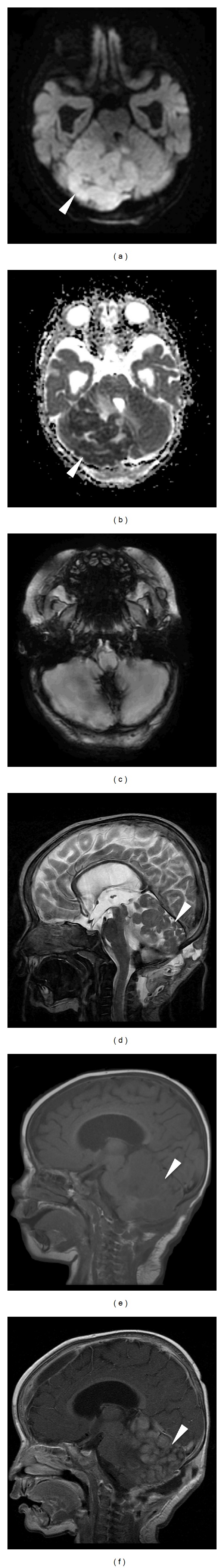
MRI features of medulloblastoma with extensive nodularity: (a)–(c) axial sequences reveal a posterior fossa mass (arrows) with decreased diffusivity on DWI (a) and ADC sequences (b), without specific intratumoral abnormalities on SWI sequence aside from amorphous susceptibility artifact present along the vermis from prior surgical biopsy (c). Sagittal sequences reveal extensive nodularity on T2 (d) and postgadolinium (f) sequences that is less evidence on precontrast T1-weighted sequences where the nodular areas appear more hypointense (e).

**Figure 2:**
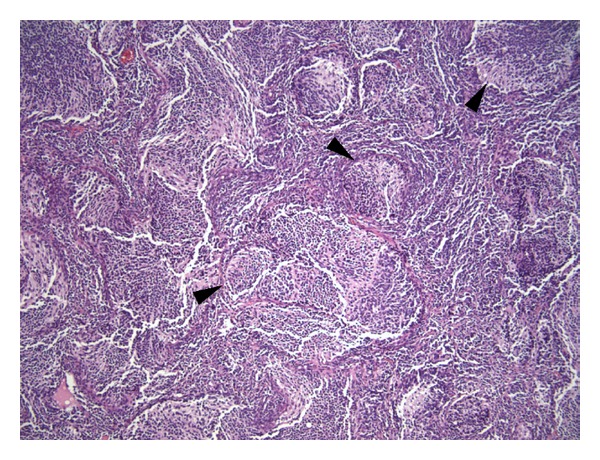
Histologic features of medulloblastoma with extensive nodularity. Hematoxilin and eosin section (4 *μ*m; 100x) reveals a malignant small blue cell tumor with widespread formation of nodules of uniform cells with neuropil formation surrounded by intervening areas of more densely populated pleomorphic cells consistent with a diagnosis of medulloblastoma with extensive nodularity. The arrowheads point to the nodular zones.

A 10-month-old full term female presented to our hospital following a 4–6 month history of delayed motor milestones and enlarging head circumference. She had an external ventricular drain placed at an outside hospital prior to arriving at our institution. Her neurological examination revealed mild limited upgaze and moderate axial hypotonia without obvious dysmetria. Magnetic resonance imaging (MRI) examination of the brain revealed a very large posterior fossa tumor with decreased diffusivity on diffusion-weighted imaging (DWI) and apparent diffusion coefficient (ADC) sequences (Figures 1(a) and 1(b)). On T2-weighted and post-gadolinium sequences, the tumor shows an extensive nodular grape-like appearance. MRI of the spine was negative for leptomeningeal spread (not shown). Given the patient's young age and unique neuroimaging characteristics, a medulloblastoma with extensive nodularity was suspected. Permanent histology revealed a small round blue cell tumor, with extensive nodular areas of densely packed reticulin-rich cells consistent with a diagnosis of medulloblastoma with extensive nodularity (Figure 2). The nodular areas have an impressive neuroradiographic correlate on both T2 and postgadolinium T1-weighted sequences (Figure 1). After surgery the patient had numerous small areas of residual disease that were scattered throughout the cerebellum. Given the favorable histology and improved examination after operation, it was decided to proceed with chemotherapy only in order to delay or avoid both another surgery and/or radiation therapy.

## 3. Discussion

The five-year overall survival of children with average risk medulloblastoma who are treated with a combination of craniospinal radiation and chemotherapy approaches 90%. Currently average risk is defined as age greater than 3 years at diagnosis, negative CSF or leptomeningeal spread, and less than 1.5 cm^2^ of residual tumor postmaximum surgical resection. The newly stratified molecular classification of medulloblastoma has revealed distinct subsets of children who have better prognosis in spite of their young age. Among these children are those with either nodular desmoplastic or extensive nodularity. The distinguishing feature at the histologic level is those patients with extensive nodularity have an expanded lobular architecture of the classic reticular free zones that are enlarged with neuropil. Previously termed “cerebellar neuroblastoma” medulloblastoma with extensive nodularity belongs to a molecular subgroup driven by sonic hedgehog pathways. The neuroradiographic features of this medulloblastoma with what we describe as “excessive” nodularity are important to recognize as these children may be cured with chemotherapy alone and spared from the neurocognitive consequences of radiation therapy. While a similar finding of medulloblastoma with “extreme” nodularity has been reported [10], we feel that our patient shows the most extensive nodularity reported to date. Furthermore we have shown an extensive area of neuroradiographic sequences including ADC, DWI, T2-weighted, and postgadolinium features along with the corresponding neuropathologic nodularity correlates. The differential diagnosis of similar grape-like characteristics of a posterior fossa mass in a patient of similar age would include atypical teratoid rhabdoid tumor, atypical choroid plexus carcinoma, and dysplastic gangliocytoma of the cerebellum or Lhermitte-Duclos disease. The distinguishing feature between Lhermitte-Duclos and medulloblastoma with extensive nodularity is the avidity for gadolinium enhancement in the latter. Our case study significantly enhances our understanding of the neuroradiographic features of this uncommon tumor. An increased understanding of the various neuroimaging features of medulloblastoma may be able to better help us substratify patients based on neuroimaging characteristics similar to what has been performed at the molecular level.

## Abbreviations

MRI: Magnetic resonance imaging
DWI: Diffusion-weighted imaging
ADC: Apparent diffusion coefficient.
